# The susceptibility of *Anopheles lesteri *to infection with Korean strain of *Plasmodium vivax*

**DOI:** 10.1186/1475-2875-8-42

**Published:** 2009-03-12

**Authors:** Deepak Joshi, Wej Choochote, Mi-Hyun Park, Jung-Yeon Kim, Tong-Soo Kim, Wannapa Suwonkerd, Gi-Sik Min

**Affiliations:** 1 Department of Biological Sciences, Inha University, Incheon, South Korea; 2 Department of Parasitology, Faculty of Medicine, Chiang Mai University, Chiang Mai, Thailand; 3 Division of Malaria & Parasitic Diseases, National Institute of Health, Seoul, South Korea; 4 Department of Parasitology, College of Medicine, Inha University, Incheon, South Korea; 5 Office of Vector Borne Diseases Control, Department of Communicable Disease Control, Ministry of Public Health, Chiang Mai, Thailand

## Abstract

**Background:**

Following its recent re-emergence, malaria has gained renewed attention as a serious infectious disease in Korea. Three species of the Hyrcanusgroup, *Anopheles lesteri, Anopheles sinensis *and *Anopheles pullus*, have long been suspected malaria vectors. However, opinions about their vector ability are controversial. The present study was designed with the aim of determining the susceptibility of these mosquitoes to a Korean isolate of *Plasmodium vivax*. Also, *An. sinensis *is primarily suspected to be vector of malaria in Korea, but in Thailand, the same species is described to have less medical importance. Therefore, comparative susceptibility of Thai and Korean strains of *An. sinensis *with Thai strain of *P. vivax *may be helpful to understand whether these geographically different strains exhibit differences in their susceptibility or not.

**Methods:**

The comparative susceptibility of *An. lesteri*, *An. sinensis *and *An. pullus *was studied by feeding laboratory-reared mosquitoes on blood from patients carrying gametocytes from Korea and Thailand.

**Results:**

In experimental feeding with Korean strain of *P. vivax*, oocysts developed in *An. lesteri*, *An. sinensis *and *An. pullus*. Salivary gland sporozoites were detected only in *An. lesteri *and *An. sinensis *but not in *An. pullus*. Large differences were found in the number of sporozoites in the salivary glands, with *An. lesteri *carrying much higher densities, up to 2,105 sporozoites in a single microscope field of 750 × 560 μM, whereas a maximum of 14 sporozoites were found in any individual salivary gland of *An. sinensis*. Similar results were obtained from a susceptibility test of two different strains of *An. sinensis *to Thai isolate of *P. vivax*, and differences in vector susceptibility according to geographical variation were not detected.

**Conclusion:**

The high sporozoite rate and sporozoite loads of *An. lesteri *indicate that this species is highly susceptible to infection with *P. vivax*. *Anopheles sinensis *appears to have a markedly reduced ability to develop salivary gland infection, whilst in *An. pullus*, no sporozoites were found in the salivary glands. Provided that the survival rate of *An. lesteri *is sufficiently high in the field, it would be a highly competent vector of vivax malaria.

## Background

*Plasmodium vivax *malaria has re-emerged in many malaria-endemic areas, where the disease was believed to have been eradicated [1]. In South Korea, it was assumed that malaria had been eradicated, since no indigenous case was reported after 1978. However, following the detection of a case in 1993, the annual incidence of malaria increased dramatically and reached a peak of 4,142 cases in 2000 [2-5]. In the meantime, malaria cases in North Korea increased from 1,085 in 1998 to 296,540 in 2001 [6]. As a result, malaria has gained renewed attention as a public health problem throughout the Korean Peninsula.

So far, eight anopheline mosquitoes have been reported from South Korea. Six of these species belong to the Hyrcanus group: *Anopheles sinensis*, *Anopheles lesteri*, *Anopheles pullus*, *Anopheles sineroides*, *Anopheles kleini *and *Anopheles belenrae *[7-10]. Two other non-Hyrcanus group species are *Anopheles koreicus *and *Anopheles lindesayi japonicus *[7].

Since vivax malaria has been recognized as an endemic disease particular to countries in the Far East Asia, including Korea, efforts to find the main vector(s) have continuously proceeded from Korea, Japan and China [7,11-13]. However, there is currently no available report regarding the vector susceptibility of the two non-Hyrcanus species and *An. sineroides*, because these species are very rare in Korea, Japan and China and, are thus considered medically less important [7,14,15]. Although there is some published literature on the two newly reported species, *An. belenrae *and *An. kleini *[9], the biting behaviour, larval habitats, and medical importance of these species are still unknown [16]. The results of older studies on the vector abilities of *An. lesteri*, *An. sinensis *and *An. pullus *are controversial. *Anopheles sinensis *has long been considered a primary and strong vector in Korea and Japan [11,12,15,17], while in countries like China and Thailand, this mosquito is treated as a refractory vector with weak transmission capability [13,18,19].

Although *An. lesteri* has been considered a minor and weak vector in Korea and Japan, studies in China showed that it was an important vector [20]. Conflicting reports on the vector ability of *An. pullus* may also be found [12,4], with some suggesting that it is a malaria vector whereas others indicate otherwise [21]. Thus, the malarial susceptibility of these vectors remains to be clarified.

In this study, three species of the Hyrcanus group, *An. lesteri, An. sinensis *and *An. pullus *were experimentally infected with a Korean isolate of *P. vivax *and the malarial susceptibility of these species was analysed based on their ability to develop oocysts in the midgut and sporozoites in salivary glands. To substantiate the findings of the above experiments and to further understand and verify the existing differences in vector abilities of two geographically distant strains of *An. sinensis *from Korea and Thailand, experimental infections were conducted with a Thai isolate, and the ability of both these strains to develop oocysts and sporozoites was determined.

## Methods

### Colonization of mosquitoes

Three mosquito lines of the Korean *Anopheles *raised in the laboratory were *An. lesteri*, *An. sinensis *and *An. pullus*. Among these, *An. sinensis *and *An. pullu*s were collected from Paju City, Gyeonggi-do Province, while *An. lesteri *was collected from So-Rae District, Incheon City in South Korea. The Thai strain of *An. sinensis *was collected from Mae Tang District, Chiang Mai Province, Thailand. *Anopheles cracens *was in a free-mating colony established for more than two decades in the insectariums of the Department of Parasitology, Chiang Mai University, Thailand and was used as a control, since it is known to be highly susceptible to *P. vivax *[22]. These colonies were reared using the method described by Park *et al *[23] in an insectary room at 27 ± 2°C, 70–80% relative humidity with 12:12 hr light and dark photoperiods adjusted by fluorescent lighting.

### Infected blood

Blood containing gametocytes of Korean *P. vivax *was supplied by the National Institute of Health (KNIH), from a patient seeking treatment at Gimpo, Gyeonggi-do province. The Thai strain of *P. vivax *was obtained from malaria patient infected in Mae Tang District of Chiang Mai Province. Informed consent was obtained from the patients before collection and the study protocols were approved by Internal Review Board of Korea National Institute of Health and Thailand Office for Vector Borne Diseases Control, Department of Communicable Diseases Control, Ministry of Public Health. Giemsa staining of the blood film was performed and gametocytes were counted. The gametocyte density of the Korean isolate of *P. vivax *was 36 gametocytes/200 White Blood Cells (WBC) while that of the Thai strain was 26 gametocytes/200 WBC.

### Infection of mosquitoes with *P. vivax *gametocytes

Three to five days old females were used for infection. To enhance their willingness to feed, the mosquitoes were starved for 12 hours prior to infection and were transferred to paper cups of size 8.5 cm in diameter and 11 cm in depth (about 50 females per cup). The mosquitoes were fed on infected blood containing gametocytes through an artificial membrane feeding technique described by Chomcharn *et al *[24]. The mosquitoes were allowed to feed for one hour.

Unfed mosquitoes were removed and the fully engorged females were carefully handled and were kept in the insectary. Mosquitoes were then fed on cotton patches, which were soaked in 5% sucrose solution and changed every day, until the mosquitoes were dissected. During infection, three lines of the Korean mosquito: *An. lesteri*, *An. sinensis *and *An. pullus *were infected with Korean isolate of *P. vivax*, while strains of *An. sinensis *from Thailand and Korea together with *An. cracens *were infected with Thai isolate of *P. vivax*.

Eight and 14 days after feeding, mosquitoes were dissected to detect oocysts in the midgut and sporozoites in the salivary glands respectively. Counting of oocysts in mosquitoes was followed by examining wet mounts of the midgut stained in 0.1% mercurochrome and freely moving sporozoites were carefully detected from salivary glands placed in a drop of phosphate buffered saline (PBS, pH 7.2).

To explore differences in densities of the salivary gland sporozoites between *An. lesteri *and *An. sinensis*, two methods were used: 1) for *An. lesteri*, micrographs were taken at 100× magnification using a camera (Motic Cam 2000) mounted on a compound microscope (Leica DM 2500) and counting of sporozoites was performed within a captured micrograph, the corresponding area of which was approximately 750 μm × 560 μm; 2) sporozoites were counted from whole salivary glands from *An. sinensis *and were compared to the sporozoite loads within a single microscopic field of *An. lesteri*. The above counting and comparative procedures were developed because in highly infected *An. lesteri*, the direct counting of several hundreds of moving sporozoites while observing through the microscope was difficult. It was sometimes impossible to count all of them from salivary glands while in *An. sinensis *any such possibilities were less and few numbers of sporozoites were directly counted by changing fields. Depending on counting, mosquitoes were graded as 1+ (1–10 sporozoites), 2+ (11–100 sporozoites), 3+ (101–1,000 sporozoites) and 4+ (above 1,000 sporozoites) [25]. The total number of +'s recorded for all infected mosquitoes was gland indexes in terms of sporozoites load.

## Results

### Infection with Korean strain of *Plasmodium vivax*

As indicated by oocysts developed in the midgut, infections were detected in all three species eight days post feeding with Korean strain of *P. vivax*. However, *An. lesteri *was highly susceptible, in that all nine mosquitoes carried oocysts (100%). Compared to *An. lesteri*, fewer percent of *An. sinesis *(87%) and *An. pullus *(83%) developed oocysts.

During 14 days dissections, all 14 dissected *An. lesteri *contained oocysts. However, fewer *An. sinensis *and *An. pullus *do so. In addition, when detached salivary glands of the infected mosquitoes were examined, nine out of 14 *An. lesteri *(64%) and only two out of 19 *An. sinensis *(11%) contained sporozoites, while the examined salivary glands of all 6 *An. pullus *lacked sporozoites (Table 1).

**Table 1:** Oocysts and sporozoites detected from the mosquitoes eight and 14 days post infection with Korean strain of *P. vivax*.

**Species**	**Sample size**	**Average no. oocyst (range)**	**Percentage with oocysts**	**Percentage with sporozoites**
8 days dissections				

*An. lesteri*	9	97.2 ± 24.1 (80–144)	100 (9/9)	ND
*An. sinensis*	15	66.8 ± 44.9 (2–136)	87 (13/15)	ND
*An. pullus*	6	38.7 ± 33.5 (21–80)	83 (5/6)	ND

14 days dissections				

*An. lesteri*	14	61.1 ± 27.2 (24–105)	100 (14/14)	64 (9/14)
*An. sinensis*	19	46.3 ± 46.0 (5–100)	68 (13/19)	11 (2/19)
*An. pullus*	6	41.7 ± 41.2 (20–100)	67(4/6)	0 (0/6)

ND = not done, salivary glands were examined only after 14 days.

**Figure 1:**
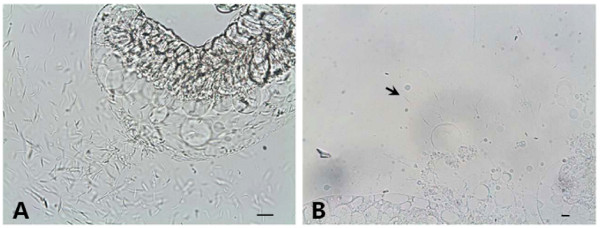
**Sporozoites detected from salivary gland of the infected mosquitoes**. (A) High number of free flowing sporozoites released from the salivary gland of *An. lesteri*. (B) Arrow pointing to one among a few number of sporozoites released from squashed salivary gland of *An. sinensis*. Dissection was done 14 days post infection. Bars = 15 μm.

Even if few numbers of *An. sinensis *contained salivary gland sporozoites, compared to *An. lesteri*, the sporozoite loads in these species were very low (Figure 1). The highest number of salivary gland sporozoites recorded from *An. sinensis *was 14. Between two positive *An. sinensis*, one had a gland index of 1+ (< 10 sporozoites) and the other had 2+ (11–100 sporozoites). While the lowest and highest numbers of sporozoites counted in one microscopic field of *An. lesteri *were 78 and 2,105. Among 9 infected *An. lesteri*, one had gland index of 2+ (11–100 sporozoites), four were with 3+ (101–1,000 sporozoites) and remaining four were with 4+ (> 1,000 sporozoites), respectively. When compared to *An. sinensis*, the lowest and highest numbers of sporozoites of *An. lesteri*, even in a single microscopic field, were 5–150 times more intense.

### Infection with Thai strain of *Plasmodium vivax*

When mosquitoes were dissected eight days after feeding with Thai strain of *P. vivax*, equal percent of *An. sinensis *from Thailand and Korea (88%) develop oocysts, except differences were seen in number of the midgut oocysts they carried. All seven dissected specimens of *An. cracens *(100%), the control species, produced higher number of oocysts compared to both strains of *An. sinensis*.

Despite 14 days of oocyst development, sporozoites were not detected from the Thai strains of *An. sinensis *while only one out of 20 specimens of Korean strain (5%) contained salivary gland sporozoites. Furthermore, salivary gland sporozoites were detected from of all *An. cracens *(100%), the control species (Table 2).

**Table 2:** Oocysts and sporozoites detected from the mosquitoes eight and 14 days post infection with Thai strain of *P. vivax*.

**Species**	**Sample size**	**Average no. oocyst (range)**	**Percentage with oocysts**	**Percentage with sporozoites**
8 days dissections				

*An. cracens*	7	69.7 ± 26.8 (39–118)	100 (7/7)	ND
*An. sinensis *(TH)	8	5.4 ± 3.1 (2–11)	88 (7/8)	ND
*An. sinensis *(KR)	8	16.3 ± 13.2 (3–40)	88 (7/8)	ND

14 days dissections				

*An. cracens*	18	NC	83 (15/18)	100 (18/18)
*An. sinensis *(TH)	14	NC	86 (12/14)	0 (0/14)
*An. sinensis *(KR)	20	NC	65 (13/20)	5 (1/20)

TH = Thailand strain, KR = Korean strain.

ND = not done, salivary glands were examined only after 14 days.

NC = Oocyst numbers not counted.

## Discussion

The abundance of *An. sinensis *in the Orient led to early suspicions of its role as a vector, but questions as to its role have continued for several decades [13,15,18]. The first successful study on experimental malarial transmission in *An. sinensis *was conducted by Tsuzuki in Japan, in 1902, which Kamimura suggested later on was actually *An. lesteri *[7]. Subsequently, Otsuru and Ohmori [15] stated that *An. sinensis *had a similar ability to develop malaria as *An. lesteri *in Japan.

Ho *et al *[13] showed that *An. lesteri*, besides being the primary vector of filarial parasites, was also the primary vector of malarial parasites in the hilly regions of the Yangtze valley (South China) and also pointed out that *An. sinensis*, due to its zoophilic behaviour, is an inefficient vector responsible for maintaining a low malaria endemicity in the broad flat rice plains of south China. Similarly, Harrison [26] opined that *An. lesteri *might well be a more efficient vector than *An. sinensis*. Lately, Liu [20] recorded a high number of infections in *Anopheles anthropophagus *(synonymized as *An. lesteri*) [27] from experimental feeding with *P. falciparum *gametocytes and also reported the natural infection rates of *An. lesteri *to be higher than *An. sinensis *in China. With such findings and along with other essential parameters (human-biting rate, human blood index, vectorial capacity and entomological inoculation rate), *An. lesteri *was considered to be 20 times more efficient as a malaria vector than *An. sinensis *in malaria infected regions in China [20,28]. In South Korea however, the first natural infections in *An. sinensis *were reported in 1962 [11]. In a similar manner, Hong [12] also reported a very low number of sporozoite positive *An. sinensis *followed by *An. pullus *(previously called *Anopheles yatshushiroensis*). These early studies supported the primary and secondary vector status of *An. sinensis *and *An. pullus*.

In recent years, much of the understanding of vector susceptibilities of Korean *Anopheles *was based on Enzyme-Linked Immunosorbent Assay (ELISA). Most of these studies indicated *An. sinensis *to be a vector [21,29,30], while recently, Lee *et al *[4] reported *An. kleini *and *An. pullus *to be stronger vectors than *An. sinensis*. Because Circumsporozoite Protein (CSP) detected by ELISA can equally be detected from developing oocysts, and sporozoites present in haemocoel, positive results from the test do not always indicate that the salivary glands are infected with sporozoites [31]. Thus, immunological evidence supporting the presence of CSP may indicate that the mosquito is a potential vector of malaria, but it is not proof that the sporozoites are located in the salivary glands and can be transmitted to a vertebrate host by a mosquito bite.

In this study, three Korean species of Hyrcanus group (*An. lesteri*, *An. sinensis *and *An. pullus*) were experimentally infected with an indigenous Korean isolate of *P. vivax*. After eight and 14 days post-infection, examination of midguts showed the presence of high number of oocysts in all three species. However, there were differences in their innate ability to develop sporozoites in the salivary glands. Sporozoites in salivary glands were detected from *An. lesteri *and *An. sinensis *but not from *An. pullus*. Though, sporozoites were detected from *An. sinensis*, they were very few as compared to *An. lesteri*. The maximum number of sporozoites in a salivary gland of *An. sinensis *was 14, which corresponded to the findings of Rongsriyam *et al *[19]. Assuming that two salivary glands have an equal number of sporozoites, fewer than 30 sporozoites are likely to develop in a pair of salivary glands of the above-mentioned mosquito. Earlier Ito *et al *[32] reported that mean densities of the sporozoites below 400 were not sufficient enough to initiate infections in mice. In such contest, low numbers of sporozoites detected from *An. sinensis *can be assumed to play less important role in initiating infections when compared to *An. lesteri*.

Because sporogony in *P. vivax *at 25°C is completed within 9 days [33], fourteen days time in this study, after which salivary glands were examined, should have been sufficient for sporozoites to reach to salivary glands. But compared to *An. lesteri*, very few number of *An. sinensis *contained salivary gland sporozoites however no sporozoites were detected in *An. pullus*. This suggests that developmental transitions couldn't proceed from oocysts to sporozoites formation in *An. sinensis *and *An. pullus*. Beier *et al *[33] described that inhibition in transitions from oocysts to sporozoites might be caused due to different mechanisms like oocysts failing to produce sporozoites, sporozoites failing to navigate successfully to the salivary glands, invading salivary glands or surviving in the salivary glands.

In addition to demonstrating sporozoites in salivary glands following laboratory infection, it is necessary to consider the natural survival rates of malaria vectors. At present, there are no connected reports with the matter in *An. lesteri*. Therefore, future studies regarding survival rate of *An. lesteri *in wild population can be more supporting evidence for this study. In Korea, the present study is the first of its kind, comparing the malarial susceptibilities of these members through successful malaria development within lab-raised clean colonies. The results are well congruent with Chinese and Thailand reports. So, the outcomes of this study have significant bearings within entire temperate regions where these species are abundant.

## Conclusion

Under laboratory conditions, salivary gland sporozoites were developed in *An. lesteri*, as readily as in the well-recognized vector. Big differences were seen in the rate and densities of sporozoites between *An. lesteri *and *An. sinensis*, whereas sporozoites were not detected from salivary glands of *An. pullus *even after 14 days of oocysts development. Also, geographically distant strains of *An. sinensis *from Korea and Thailand were similar in their ability to support malaria development. Therefore, *An. lesteri *is highly susceptible to *P. vivax *malaria as compared to *An. sinensis *and *An. pullus*.

Thus, *An. lesteri *is a potential malaria vector and its presence may be described as an under-rated public health threat. However, comparative susceptibility of the remaining members of the Hyrcanus group will be important in future to understand their role in malaria epidemiology in Korea.

## Competing interests

The authors declare that they have no competing interests.

## Authors' contributions

MGS designed the present study. Prior to this experimental work, WC facilitated DJ and MHP for training on malaria susceptibilities and improved lab colonization of *Anopheles *mosquitoes. DJ and MHP conducted all experimental studies under the supervision of MGS and WC. Malaria-infected blood was obtained from WC, WS, TSK and JYK. DJ drafted the manuscript with MHP. All the authors read the manuscript.
